# Liquid levothyroxine formulations in patients taking drugs interfering with L-T4 absorption

**DOI:** 10.3389/fendo.2022.1080108

**Published:** 2022-12-06

**Authors:** Elisa Gatta, Francesca Bambini, Caterina Buoso, Maria Gava, Virginia Maltese, Valentina Anelli, Andrea Delbarba, Ilenia Pirola, Carlo Cappelli

**Affiliations:** Department of Clinical and Experimental Sciences, Endocrine and Metabolic Unit, University of Brescia, Azienda Socio-Sanitaria Territoriale (ASST) Spedali Civili di Brescia, Brescia, Italy

**Keywords:** levothyroxine, liquid levothyroxine, softgel capsules, interfering drugs, levothyroxine malabsorption

## Abstract

**Purpose:**

To describe the current knowledge on thyroid hormonal profile in patients on liquid L-T4 therapy and drugs known to interfere with L-T4 absorption.

**Methods:**

A PubMed/MEDLINE, Web of Science, and Scopus research was performed. Case reports, case series, original studies and reviews written in English and published online up to 31 August 2022 were selected and reviewed. The final reference list was defined based on the relevance of each paper to the scope of this review.

**Results:**

The available data showed that novel levothyroxine formulations circumvent gastric pH impairment due to multiple interfering drugs such as proton pump inhibitors, calcium or iron supplements, sevelamer, aluminum/magnesium hydroxide and sodium alginate.

**Conclusion:**

New formulations can be taken simultaneously with drugs interfering with L-T4 absorption, in particular liquid formulations. Softgel capsules need more studies to support these data.

## Introduction

Thyroid hormones (TH) have a critical role in human homeostasis, having an impact on a wide array of tissues including the brain, heart, muscle, and bone and, if they are reduced or absent, they must be replaced in order to quickly restore euthyroidism.

Natural thyroid preparations, such as desiccated or thyroid extract, were the only available treatment until 1950, and contained both thyroxine (T4) and triiodothyronine (T3). After levothyroxine (L-T4) synthesizing, tablet formulations were distributed worldwide (1). At the beginning of the 2000s, liquid formulations started to be produced as drops, unit-dose ampoules or softgel capsules containing L-T4 dissolved in different solutions (2).

L-T4 absorption mainly occurs in the jejunum and ileum (3) and is maximal when the stomach is empty, demonstrating how gastric acidity has a key role in this process (4); for this reason, L-T4 tablets have to be administered with an empty stomach at least one hour before breakfast or at bedtime (5, 6). It is well known that higher daily doses of L-T4 are required in patients with jejunoileal bypass surgery or other bowel resection after surgery (7–9), gastrointestinal disorders such as coeliac disease, *Helicobacter pylori* infection and atrophic gastritis (4). Additionally, also dietary fiber (10) and espresso coffee (11) can reduce the bioavailability of L-T4. More important, many drugs, such as proton pump inhibitors (PPI) (12), calcium carbonate and ferrous sulphate supplementation (13–15), bile acid sequestrants, cholestyramine (16, 17), sucralfate (18–20), aluminum hydroxide (21) and phosphate binders (22), can significatively reduce L-T4 tables absorption when concomitantly administered.

L-T4 has a narrow therapeutic index (23); a tailored therapy is advocated in order to maintain serum thyroid stimulating hormone (TSH) levels in the normal range, avoiding iatrogenic complications or hypothyroidism symptoms (24). However, cross-sectional studies show that 30–50% of L-T4 users have an abnormal serum TSH level, mainly in patients taking multiple drugs (25–27).

This is a “*Giano bifronte*” aspect. On the one hand, the daily L-T4 dose must be increased to run behind serum TSH levels, on the other hand, withdrawing interfering drugs can develop iatrogenic hyperthyroidism. This is of particular interest in older patients (≥ 65 years old) for the well-documented increased risk of developing heart disease, osteoporosis, bone fracture and cognitive impairment (28–32).

Recently, it has been demonstrated that new formulations (liquid and softgel capsules) can circumvent the problem of incomplete absorption of L-T4 caused by changes in gastric pH due to their administration at breakfast (33–39) or after bariatric surgery (40). In addition, an increasing number of reports have also shown that these drugs can be administered with many of the aforementioned interfering drugs (33, 41–51).

The present review aimed to evaluate the thyroid hormonal profile in patients on liquid L-T4 therapy and drugs known to interfere with L-T4 absorption.

## Methods

The review was conducted according to the PRISMA statement, and the checklist is reported as **Supplementary file 1**.

A PubMed/MEDLINE, Web of Science and Scopus research was performed, for free-text words and terms related to “levothyroxine”, “liquid”, “oral solution”, “softgel capsules”, “drug interaction”, “drug interfering with L-T4 absorption”, “L-T4 malabsorption”, “proton-pump inhibitors”, “sucralfate”, “bile acid sequestrant”, “cation exchange inhibitors”, “calcium salts”, “ferrous sulfate”, “oral bisphosphonates”, “phosphate binders”. Case reports, case series, original studies and reviews written in English and published online up to 31 August 2022 were selected and reviewed.

The final reference list was defined based on the relevance of each paper to the scope of this review.

## Results

**Figure 1:**
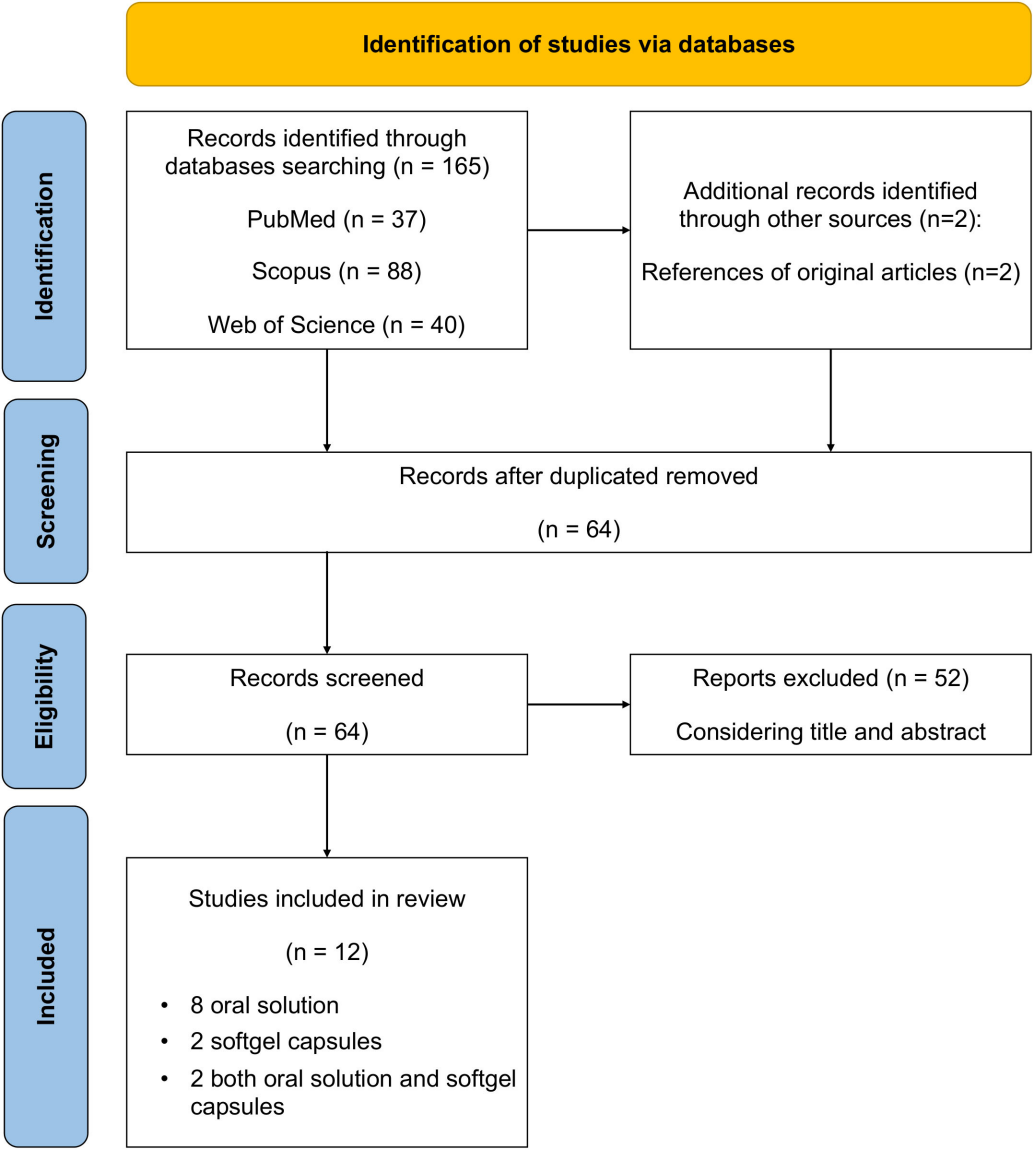
PRISMA flowchart.

In the preliminary search, 165 studies were identified through the literature search and 64 remained after the duplicates were removed. A title and abstract review was performed on the remaining studies, with 52 excluded at this first stage. A total of 12 articles were eligible for full-text screening and 12 full-text publications were included in the analysis. A PRISMA flow diagram of the screening and selection process can be found in **Figure 1**. 10 studies included in the analysis were observational, 7 of them had a prospective design and 3 were retrospective; 1 study was a double-blind crossover clinical trial; 1 was a case report. The studies were published between 2012 and 2019, all studies were from Italy.

Finally, the studies were divided according to the formulation used (liquid or softgel L-T4).

### Liquid L-T4

Ten articles regarding liquid L-T4 formulations and interfering absorption drugs were retrieved, for a total amount of 1626 patients, as shown in **Table 1**.

**Table 1:** Summary of clinical studies and case reports about liquid L-T4 formulations and interfering absorption drugs.

Author, date	Study design	Patients	Interfering drugs	Outcome
Saraceno, 2012 (51)	Prospective observational study	15	PPI	Lower serum TSH levels postswitch to liquid L-T4
Vita, 2014 (41)	Prospective observational study	24	PPI	Lower serum TSH levels postswitch to liquid L-T4
Benvenga, 2014 (42)	Prospective observational study	19	Calcium carbonate and/or ferrous sulfate	Lower serum TSH levels postswitch to liquid L-T4
Brancato, 2014 (45)	Retrospective observational study	19	PPI, calcium carbonate, aluminum hydroxide and ferrous sulphate	Lower serum TSH levels postswitch to liquid L-T4
Cappelli, 2016 (33)	Double-blind crossover clinical trial	12	PPI, calcium or iron supplements, oral anticoagulant	Lower serum TSH levels postswitch to liquid L-T4
Vita, 2017 (43)	Prospective observational study	11	PPI, calcium or iron supplements, sevelamer, aluminum/magnesium hydroxide and sodium alginate	Lower serum TSH levels postswitch to liquid L-T4
Ferrara, 2017 (46)	Retrospective observational study	1175	Multiple interfering drugs	Lower serum TSH levels postswitch to liquid L-T4
Guglielmi, 2018 (44)	Retrospective observational study	322	Multiple interfering drugs	Lower serum TSH levels postswitch to liquid L-T4
Morini, 2019 (48)	Prospective observational study	9	Calcium carbonate	Lower serum TSH levels postswitch to liquid L-T4
Benvenga, 2019 (49)	Prospective observational study	20	Multiple interfering drugs	Lower serum TSH levels postswitch to liquid L-T4

In a prospective non-randomized study, Vita R et al. enrolled 24 patients in PPI and L-T4 tablets therapy. The patients were switched to liquid oral solution maintaining the same L-T4 dosage and interval before the PPI; after 8 and 16 weeks, TSH values were checked. The study showed, for the first time, that patients in whom LT4 tablets failed to normalize or suppress the TSH serum because of the concomitant ingestion of PPI benefited from the switch from tablet to oral solution (41).

Data were confirmed by Saraceno G et al., demonstrating that liquid formulation can solve the problem of incomplete absorption caused by PPI in 15 patients on therapy with different PPIs (51).

The same Authors provided the same results in 19 patients taking calcium carbonate, ferrous sulfate or both (42).

In 2017, Vita R et al. demonstrated the capability of liquid solution to circumvent gastric pH impairment due to multiple interfering drugs (IPP, calcium or iron supplements, sevelamer, aluminum/magnesium hydroxide and sodium alginate) in 11 patients. The switch from L-T4 tablets to liquid permitted reaching the TSH target already after 8 weeks. In detail, mean TSH values under tablet L-T4 were 4-fold higher compared to those under liquid L-T4 (4.3 ± 3.1 vs. 1.1 ± 1.3 mU/L, P< 0.0001) (43).

Guglielmi V et al. conducted a real-life study in primary care, identifying 3965 patients that were taking drugs which interacted with L-T4 absorption, as recorded in the Italian general practice Health Search IMS Health Longitudinal Patients Database (HSD). 322 patients (8%) took liquid L-T4, whereas 3643 (92%) tablets. Serum TSH levels were higher in the latters (2.15 mU/L versus 1.82 mU/L). The Authors concluded that, in clinical practice, the use of oral liquid LT4 is not associated with the increase in daily dosage of L-T4 compared with tablet formulation during exposure to interfering drugs (44).

An observational retrospective study enrolled 53 patients on L-T4 replacement therapy who switched from L-T4 tablets to liquid formulation without changing the daily dose. Among them, 18 (34%) were taking drugs that interfered with LT4 absorption (PPI, calcium carbonate, aluminum hydroxide and ferrous sulphate). The ratio between serum TSH levels 60-90 days postswitch and preswitch were 0.50 (0.31-0.72; 95% CI 0.33-0.69) (45).

Ferrara R et al. conducted a longitudinal real-world study collecting data from 56,354 patients in L-T4 treatment. Among them, 19044 (34%) subjects were also taking drugs which interacted with L-T4 absorption; 501 switched to oral drops and 674 to pre-dosed vials. The Authors demonstrated a significant TSH reduction with respect to preswitch, both in drops and pre-dosed vials group (46).

Our group conducted a double-blind placebo-controlled trial, enrolling 77 naive hypothyroid patients in order to demonstrate if an oral solution of L-T4 could be ingested during breakfast. We observed no influence of breakfast composition and co-treatment with other drugs (in 11 patients, including PPI) on TSH levels (33).

### Softgel capsule L-T4

Four articles regarding softgel L-T4 formulation capsules and interfering absorption drugs were retrieved, for a total amount of 47 patients, as shown in **Table 2**.

**Table 2:** Summary of clinical studies and case reports about softgel capsules L-T4 formulations and interfering absorption drugs.

Author, date	Study design	Patients	Interfering drugs	Outcome
Benvenga, 2012 (50)	Observational prospective study	19	PPI	Better absorption of softgel L-T4
Vita, 2014 (47)	Case report	1	PPI	Lower serum TSH levels postswitch to softgel L-T4
Morini, 2019 (48)	Observational prospective study	7	Calcium carbonate	Lower serum TSH levels postswitch to softgel L-T4
Benvenga, 2019 (49)	Observational prospective study	20	Multiple interfering drugs	Lower serum TSH levels postswitch to softgel L-T4

Vita R et al. described a 48-years old woman in whom the impaired absorption of the levothyroxine (L-T4) tablet due to a proton pump inhibitor (PPI) use was corrected by switching the patient to the softgel capsules. Intestinal absorption of L-T4 was later evaluated by administering 600 mg of LT4 as a tablet or softgel capsule while maintaining pantoprazole; pharmacokinetic indices showed better and faster absorption of the softgel capsules versus tablets, at least in this patient (47).

This was confirmed in a randomized crossover study including 16 healthy volunteers. An acute loading with 600 μg of L-T4 (tablets *versus* softgel capsules) repeated after an acute infusion of 80 mg esomeprazole was conducted. The absorption of L-T4 softgel capsule was significantly increased at baseline and not affected by overload of PPI (50).

## Discussion

Several pathological conditions and endogenous and exogenous factors are well known to interfere with tablet L-T4 pharmacokinetics (52). In particular, various frequently used medications, such as proton pump inhibitors (PPIs), ferrous sulphate, sucralfate, raloxifene, bile acid sequestrants, calcium carbonate, phosphate binders, and aluminum-containing antiacids have been demonstrated to interfere with LT4 absorption, either by increasing gastric pH (the case of PPIs) or binding LT4 into insoluble complexes (the case of calcium or iron salts) (4).

This is well documented for tablet formulation, which needs to be dissolved in the acid gastric pH prior to its absorption at the levels of the duodenum and jejunum. In fact, gastric acidity plays an important role in the absorption of L-T4, as has also been shown by Centanni et al. (53). The mechanism by which intestinal absorption of L-T4 may be impaired in patients with altered gastric pH is still unclear. However, it is possible that hydrophilic sodium salt (pharmaceutical L-T4) remains undissociated in hypochlorydic gastric conditions, thus being absorbed less (54). For these reasons, current guidelines for the treatment of hypothyroidism by a Task Force of the American Thyroid Association recommend that, for optimal and consistent absorption, L-T4 should be taken in a fasting state away from interfering drugs (55).

In the last 20 years, pharmaceutical Companies have developed new L-T4 formulations in liquid solutions. Liquid formulations don’t need to dissolve, as they do not require a gastric acid environment. For these reasons, it reaches plasmatic maximum concentrations (C_max_) quicker and the median C_max_ is higher than tablets with a higher gastrointestinal absorption (40, 56–58). This is well evidenced by Brancato et al. and Negro et al. that observed a significant decrease in TSH serum levels in patients previously on therapy with L-T4 tablets after switching to liquid L-T4 (45, 59). These data were confirmed in patients taking liquid L-T4 concomitantly with breakfast (33, 34, 36, 37, 40) and this can improve patients’ compliance and quality of life (38, 60). In addition, it has been demonstrated that novel L-T4 formulations are more effective in reaching normal serum TSH levels in patients who underwent bariatric surgery (40, 61) or with autoimmune gastritis (62), lactose intolerance (63), *Helicobacter pylori* infection (64), giardiasis (65), diabetic gastroparesis (66), esophageal complications of systemic sclerosis (67) or liver cirrhosis (68), all conditions that somehow impair gastric acidity.

Softgel capsules may represent combinations of practicality of the tablets formulations and pharmacokinetics qualities of liquid ones, resulting in a better gastrointestinal absorption than tablets (56, 57, 69). In fact, changes in intraluminal gastric pH have a negligible effect on liquid and softgel capsules L-T4 absorption (70, 71). In agreement we previously showed that softgel formulations of L-T4 can be taken with breakfast (35), data confirmed by few authors (72, 73).

Levothyroxine is the second prescribed drug in the United States (74). It is important to underline that most of the molecules known to interfere with gastric L-T4 absorption are, at the same time, among those most prescribed (75). For this reason, it is quite common for a patient to be on concomitant treatment with L-T4 and drugs interfering with its absorption. This was well evidenced by Benvenga et al., that showed PPIs as the most frequent molecules either alone (74.8%) or in combination (25.2%) with other interfering drugs (76). Trifirò and colleagues confirmed and extended this result in a large set of patients who required higher L-T4 daily doses when PPIs were co-administered (77). On the other side, data obtained in a large series of patients on liquid L-T4 have clearly demonstrated that this formulation circumvents the co-administration of interfering drugs (41–46, 51). This was confirmed in a subset of patients enrolled in our prospective double-blind placebo study (33). Promising data are available also for softgel capsules (47, 50). Particularly, Benvenga conducted a prospective study showing that 95% of patients taking softgel capsules reached euthyroidism, even in presence of interfering drugs (49). This data were confirmed by Morini et al. (48). More studies in a larger cohort of patients are needed to confirm this important point on softgel capsules.

## Conclusion

We summarized the available reports and studies regarding liquid and softgel L-T4 formulations and drugs known to interfere with levothyroxine absorption.

New formulations can be taken simultaneously with drugs interfering with L-T4 absorption, in particular liquid formulations. Softgel capsules need more studies to support these data.

## Author contributions

CC conceived the project. CC, AD, and EG provided supervision and project administration. EG, FB, MG, CB, VM, and VA did the literature search. EG wrote the original draft. CC and IP reviewed and edited the manuscript. All authors contributed to the article and approved the submitted version.
